# Safety and feasibility of transcutaneous vagus nerve stimulation in mild cognitive impairment: VINCI-AD study protocol

**DOI:** 10.1186/s12883-023-03320-5

**Published:** 2023-08-02

**Authors:** Helena Dolphin, Adam H. Dyer, Tim Dukelow, Ciaran Finucane, Sean Commins, Sean P Kennelly

**Affiliations:** 1 Department of Medical Gerontology, Trinity College, Dublin 2, Dublin, Ireland; 2 Institute of Memory and Cognition, Tallaght University Hospital, Dublin 24, Tallaght, Ireland; 3 Department of Geriatric Medicine, Cork University Hospital, Cork, Ireland; 4 Department of Medical Physics, St James’s Hospital, Dublin, Ireland; 5 Department of Psychology, Maynooth University, Maynooth, Ireland; 6 Age-Related Healthcare Department, Tallaght University Hospital, Tallaght, Ireland

**Keywords:** Vagus nerve stimulation, Cognitive dysfunction, Nerve stimulation, transcutaneous, Autonomic Nervous System, Mild Cognitive Impairment

## Abstract

**Background:**

Over 55 million adults are living with dementia globally, which is projected to reach 157 million by 2050. Mild cognitive impairment (MCI), a syndrome of memory impairment with intact activities of daily living, may precede dementia by several years. Around 5–15% of individuals with MCI convert to dementia annually. Novel treatments which delay progression of MCI to dementia are urgently needed. Transcutaneous vagal nerve stimulation (tVNS) is a non-invasive neuromodulation technique that targets the vagus nerve. Importantly, tVNS has been shown to improve cognition in healthy volunteers, but has not been extensively examined as a potential therapeutic approach in MCI. VINCI-AD will examine the safety and feasibility of tVNS in older adults with MCI.

**Design:**

VINCI-AD is an investigator-led, single-site, single-blind, sham-controlled crossover pilot study which aims to assess the safety and feasibility of tVNS in 40 participants with amnestic MCI. All participants will attend for three consecutive study visits during which they will be randomised to receive no stimulation (baseline), active tVNS stimulation (stimulation at cymba conchae of left ear) or sham tVNS stimulation (at earlobe). Safety will be primarily assessed by ascertainment of adverse events. Further safety assessment will examine the impact of acute tVNS on subjective (orthostatic symptoms), peripheral (finometry-based blood pressure) and central (assessed via Near Infrared Spectroscopy [NIRS]) haemodynamic responses to active stand. Feasibility will be determined using a custom-designed occupational assessment of device usability. Exploratory secondary analysis in VINCI-AD will examine the potential impact of acute tVNS on associative memory, spatial memory and inhibitory control to inform sample size estimates for future trials of tVNS in older adults with MCI.

**Discussion:**

VINCI-AD will report on the safety (adverse events/haemodynamic responses to active stand) and feasibility of tVNS as a potential therapeutic option in MCI. Detailed reporting of study eligibility and completion rates will be reported. Exploratory analysis will examine the potential cognitive benefits of acute tVNS on cognitive function in MCI to report potential effect sizes that may inform future clinical trials in this cohort.

**Trial registration:**

https://clinicaltrials.gov/ct2/show/NCT05514756. Trial Registration Number NCT05514756 (24th August 2022 for this protocol, version 1.0.)

**Supplementary Information:**

The online version contains supplementary material available at 10.1186/s12883-023-03320-5.

## Background

Mild Cognitive Impairment (MCI) is a clinical syndrome characterised by cognitive impairment and deficits on neuropsychological testing, with intact activities of daily living [1, 2]. Typical estimates suggest a 5–15% conversion rate to dementia per year in those with MCI [4, 5]. Targeting individuals at high-risk for dementia, such as those with MCI, may represent an important strategy for non-pharmacological interventions targeted at dementia prevention. One such approach which has shown promise in MCI is through non-invasive brain stimulation [3].

Vagus Nerve Stimulation (VNS) involves electrically stimulating the vagus nerve (VN) either directly using implanted VNS (iVNS) devices to directly stimulate the cervical VN or using novel transcutaneous VNS (tVNS) devices. Importantly, tVNS devices are portable, handheld, relatively inexpensive and do not require surgery for implantation. tVNS devices pulse electricity gently through the skin in specific areas (such as the cymba conchae of the external ear) which have extensive VN afferent branches [4]. This has been shown to modulate subcortical and cortical activity even with relatively short burst of stimulation and may have beneficial effects on cognitive function [5–8].

Improved cognition has been noted in populations treated with iVNS for epilepsy and depression since its approval by the FDA [9–16] A one -year pilot study found that iVNS devices stabilised or improved cognitive performance in participants with Alzheimer’s disease [17, 18]. In non-clinical populations, tVNS has been shown to exert cognitive benefits across several domains including emotional recognition, post-error slowing and lexical fluency [19–26]. However the most consistent cognitive improvement in healthy populations based on meta-analytic evidence has been for executive function [27].

Older adults with MCI and dementia are at increased risk for Neuro-Cardiovascular Instability (NCVI) and Orthostatic Hypotension (OH) [28, 29]. NCVI/OH may even influence clinical progression and mortality in those with cognitive impairment and dementia [30, 31]. The VN is the primary mediator of neuro-cardiovascular reflexes including the baroreceptor response that maintains cerebrovascular perfusion during systemic hypotension [35]. Given the prominent role of the VN in NCVI, it is crucial to understand the effect of VN modulation on NVCI phenotypes in older adults with MCI, who may be at greater risk of OH and other NCVI phenotypes. Whilst VN stimulation may exert beneficial effects on NCVI, it is of paramount to establish its safety in older adults with MCI. Key measures to assess the neuro-cardiovascular response to acute tVNS stimulation include: measurement of Heart Rate Variability (HRV) – a marker of VN activity, peripheral (finometry based beat-to-beat blood pressure assessment) and central (assessed via Near Infrared Spectroscopy [NIRS]) responses to active stand.

Vagally-mediated transient reductions cerebral perfusion in those with MCI could be deleterious, as those with MCI have reduced cerebral blood flow compared to Cognitively Unimpaired (CU) individuals at baseline [32]. tVNS, however, specifically exploits the peripheral anatomy of the VN, and it is the right cervical VN that provides most negatively chronotropic effects via the sinoatrial node. Hence only the cymba conchae of the left ear is stimulated during tVNS, as the left VN provides cardiac innervation via the atrioventricular node which is less likely when stimulated to cause bradycardia [33, 34].

Oxygenation of cerebral tissue can be noninvasively measures using tools such as NIRS devices. NIRS devices project near-infrared light into underlying tissue (such as cerebral cortical tissue) and measures the backscatter of this light using detectors. Based on the amount of backscatter, the concentration of oxygenated haemoglobin and deoxygenated haemoglobin can be determined and from this information the Tissue Saturation Index (TSI; a measure of tissue oxygenation) can be determined [35]. The TSI has emerged as an objective surrogate marker of cerebral perfusion, suitable for clinical testing of dynamic cerebrovascular hemodynamics during standing [36] and has been evaluated as a marker of oxygenation during tVNS and cognitive testing as well [37]. There is a known age-related reduction in resting-state TSI in the prefrontal cortex in older adults [38] and persons with MCI have lower resting state TSI levels compared to healthy controls [39, 40]. Whether frontal TSI could be augmented by tVNS in a population with MCI remains to be explored.

The Vagus Nerve Stimulation in Mild Cognitive Impairment (VINCI-AD) study will examine the use of an easy-to-use, tVNS device in MCI. VINCI-AD will determine the safety (adverse events ascertainment, peripheral and central haemodynamic responses to active stand) and feasibility of tVNS as a potential treatment in older adults with MCI. Pre-specified exploratory secondary analysis will assess the impact of acute tVNS on cognitive function (namely associative memory, spatial memory, inhibitory control, language and working memory) to provide potential effect size estimates to inform future clinical trials of tVNS in MCI.

## Methods/design

### Study design

**Fig. 1:**
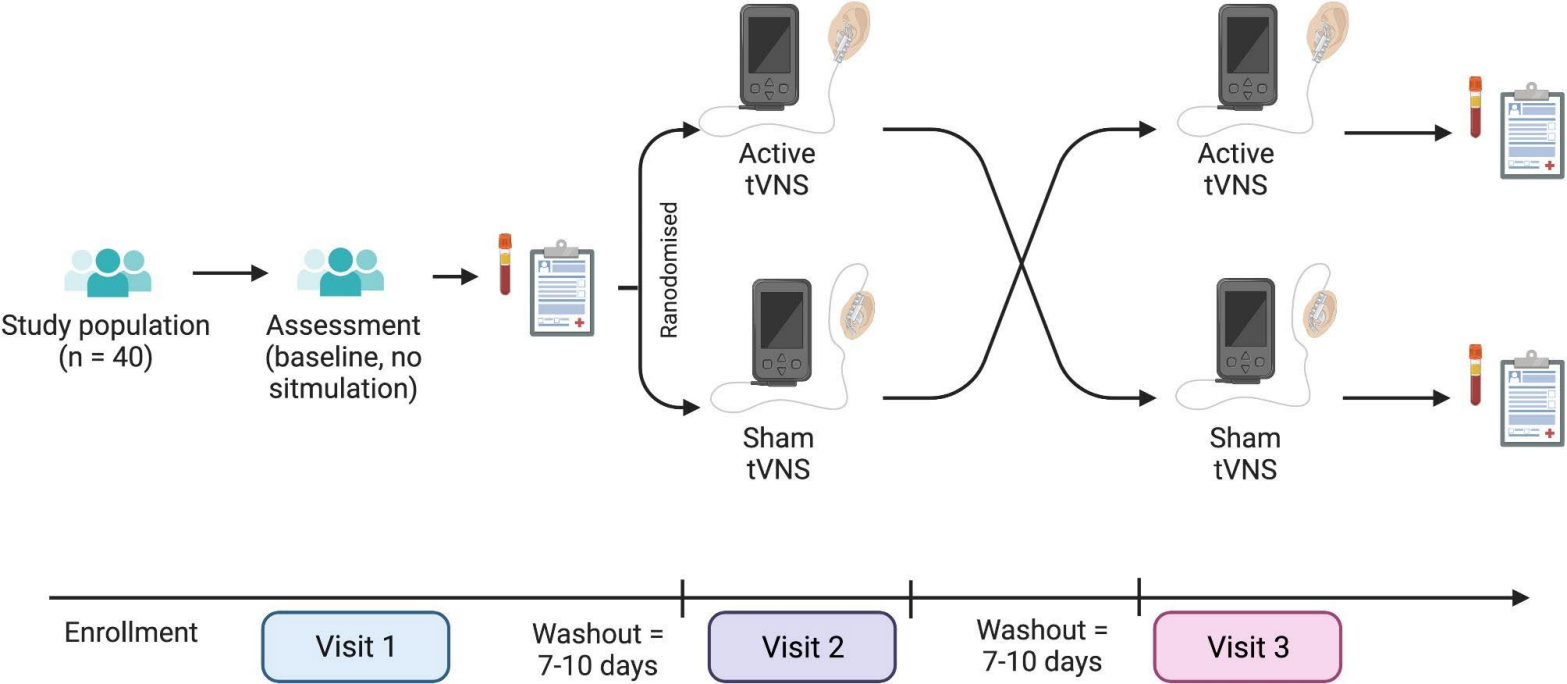
Method of crossover

VINCI-AD is a single-site, single-blind, sham-controlled crossover pilot study in patients with amnestic MCI. Each participant is assessed at baseline (no stimulation) followed by randomisation to both sham stimulation and active tVNS stimulation over two subsequent study visits. The study comprises a baseline assessment (visit 1) followed 7–10 days later by either an active or sham stimulation study visit and again 7–10 days later by a third visit wherein the final condition (active or sham) will be undertaken. The inclusion of a sham stimulation condition is to confound for potential unblinding, which is common in device trials. Participants will be enrolled and consented by study clinician (HD). Please see Fig. 1 for an illustration of randomisation and sham stimulation technique.

Our study will follow the Standard Protocol Items: recommendations for Interventional Trials (SPIRIT) guidelines [41]. Table 1 illustrates the SPIRIT flow diagram summarising study procedures, outcome measures, randomisation and assessments for each study visit.

**Table 1:** Flowchart of VINCI-AD Protocol Timeline

			Study Period					
			Screening (day − 90 to day − 7)	Visit 1 (day 0)		Visit 2 (day 7–10)		Visit 3 (day 14–17)
Enrolment								
- Eligibility Screen			x					
- Informed Consent			x					
Intervention								
- Baseline assessments (no stimulation)				x				
- Active (left cymba conchae) stimulation						ø		ø
- Sham (left earlobe) stimulation						†		†
Assessments								
- General assessment, vitals, medication changes				x		x		x
- Primary Outcome: Safety								
- Assess for AE/ SAEs						x		x
- Active stand with TSI				x		x		x
- Primary Outcome: Acceptability and Tolerability								
- Likert scale questionnaires						x		x
- Cognitive assessments								
- FNAT				x		x		x
- SART				x		x		x
- SHQ				x		x		x
- Verbal word learning				x		x		x
- Semantic fluency				x		x		x
- Digit span forwards				x		x		x
- Digit span backwards				x		x		x
- Verbal word recall				x		x		x
- Verbal word recognition				x		x		x
- Frontal Oxygenation								
- Cognitive testing TSI				x		x		x
- Neurocardiovascular assessments								
- HRV				x		x		x
- Serum / Plasma venepuncture								
- Inflammatory cytokine panel				x		x		x
- Device usability assessment								
- Kettle test								x

Ø = active stimulation randomisation: participant randomised to either sham or active stimulation at Visit 2 and alternative stimulation site at Visit 3

† = sham stimulation randomisation: participant randomised to either sham or active stimulation at Visit 2 and alternative stimulation site at Visit 3

AE – Adverse event; SAE – Serious adverse event; FNAT – Face Name Association Task; SART – Sustained Attention Response Task; SHQ – Sea Hero Quest Tas; NIRS – functional Near Infrared Spectroscopy; HRV – Heart Rate Variability; TSI – Tissue Saturation Index

### Study setting

This pilot study is carried out at the Institute of Memory and Cognition, Tallaght University Hospital, Dublin, Ireland which is a tertiary referral centre at a University teaching hospital. The Institute is coordinated by Consultant Geriatricians and a Consultant Neurologist with consensus diagnosis for cognitive impairment undertaken with specialised Advanced Nurse Practitioner, Occupational Therapist, Speech and Language Therapist and Neuropsychologist input.

### Eligibility criteria

VINCI-AD will recruit both males and females with a diagnosis of amnestic MCI as determined by consensus diagnosis at the Institute of Memory and Cognition. Those eligible for inclusion in VINCI-AD will have an index delayed memory score on the Repeated Battery of Neuropsychological Status (RBANS) of less than 85 (indicative of amnestic features) and a score of 0.5 on the Clinical Dementia Rating Global scale indicating mild impairment not impacting daily function. All participants mut be on stable medications for one month prior to recruitment, must have sufficient understanding of English language and must be able to provide informed consent. VINCI-AD exclusion criteria include:

1. Significant current depression
2. Uncorrected vision/hearing loss
3. History of brain surgery
4. History of epilepsy with any seizure event in last year
5. Prescribed any pharmacological agents known to significantly increase seizure risk
6. Diagnosed arrhythmia including atrial fibrillation
7. Cardiac pacemaker implant
8. Existing left ear deformity or recent ear trauma
9. Alcohol dependence
10. Prescribed DMARDs or immunotherapies

### Assessment procedures

Participants will undergo a baseline visit to assess eligibility by the study clinician (HD). This baseline visit will include a detailed clinical review to encompass clinical, demographic and anthropomorphic data, cognitive history and medication review (please see Additional File 1 for baseline assessment proforma).

### Intervention

Participants will receive active and sham stimulation with the NEMOS © CerboMed tVNS device. Two small electrodes emit a low-voltage electric signal which permeates the skin. When the electrodes are placed at the cymba conchae of the ear, stimulation actives the afferent branch of the VN that innervates the skin of the ear (Arnold’s nerve). The electric signal consists of an 8 Hz frequency wave with participant-selected amplitudes ranging from 0.1 mA to 5 mA depending on their sensory threshold, the signal cycles of 200 ns and comes in 30 s on – 30 s off bursts. For the sham condition the electrode is placed on the earlobe which is free of vagus innervation. For the active condition it is placed on the cymba conchae of the left ear. At each stimulation study visit the device is explained to the participant and then fitted by the study researcher. Participants are given oral and written instructions at each stimulation study visit regarding how to set up and fit the device in their ear.

### Safety and tolerability of tVNS in MCI

The risk associated with use of the NEMOS © Cerbomed tVNS device is minimal as published data of the safety profile shows minimal adverse events. The most common adverse events reported include local skin tingling, burning, redness, itching, tinnitus or headache which occur in up to 18% of participants, and tends to resolve quickly after discontinuation of the use of the device [42]. Due to the theoretical risk of increasing cardiac vagal-mediated bradycardias or hypotensive episodes by stimulating the right VN, participants will have stimulation of the left ear only.

During the active and sham stimulation conditions, safety will be recorded by completion of the following:

- Contemporaneous recording of any adverse events reported by participants whilst undergoing either active or sham stimulation.
- Likert-Based Questionnaires (See Additional File 2) will assess for established side effects previously reported with tVNS: pain, skin discomfort, tingling, headache and tinnitus. These will be administered both during active and sham stimulation.
- Detailed assessment of subjective (OH symptoms including faintness, light-headedness), peripheral (beat-to-beat blood pressure) and central (TSI) haemodynamic responses to active stand.
  - Briefly, participants will undergo active stand from supine to standing. After lying supine for 5 min, participants will be asked to stand as fast as they can. Beat-to-beat blood pressure and heart rate responses (assessed via the Finapres© NOVA) will be monitored during active stand and for three minutes after. To standardise assessments, all assessments are all taken at the same time each day, and all routine medications are taken as usual each morning. OH is defined by consensus as a sustained reduction of systolic blood pressure (SBP) of at least 20 mmHg or diastolic blood pressure (DBP) of 10 mmHg within 3 min of standing [43].
- During active stand, cerebral oxygenation (TSI) will be estimated using NIRS-based analysis software. TSI analysis will be undertaken using the PortaLite OxySoft© NIRS device (PortaLite, Artinis Medical Systems B.V., Elst, The Netherlands) and the light emitting diode will be placed on clean skin on the right forehead, devoid of moisture or oil, and secured with a black headband. TSI readings are transferred via Bluetooth in real-time and will give an approximation of tissue oxygenation during rest, active stand, and all cognitive assessments.

Following assessment, participants will be given contact details of the principal investigator and study coordinator at the hospital who they can contact if they have concerns, or any new symptoms arise. Any adverse events will be diarised during the intervention and closely monitored until resolution. Data of participants who discontinue or deviate from the study protocol will be retained for intention to treat analysis.

### Feasibility and usability assessment

To assess feasibility of tVNS in MCI, VINCI-AD will assess report the number of participants completing assessment across the three sessions. To assess usability of the device, a modified Kettle test has been designed with senior Occupational Therapy colleagues in our institution (see Additional File 3). The Kettle test is a brief, objective assessment of functional skills [44] which has been designed specifically to ascertain the participants’ ability to independently use the NEMOS© tVNS device. At the end of the third session whereby the participants will have been introduced to the NEMOS© tVNS device on three occasions, worn it for two sessions (one active at the cymba conchae and one sham at the earlobe), they will then be presented with the device and asked to set up and use the device as independently as possible. There are 13 objective tasks detailed and each is graded by the assessor from 0 (no assistance required, fully independent) to 4 (fully assisted with this aspect of task performance). Scores on this task range from 0 to 52, with lower scores indicating more independent use of the device and higher scores reflecting more severe difficulty in using the device independently.

Acceptability of the device will be assessed via Likert-based scale questions including if the device felt comfortable, their subjective sense of confidence using the device, would the participant use it again (See Additional File 4).

### Pre-specified exploratory analysis

#### Potential effects of acute tVNS on cognitive performance

VINCI-AD is a safety and feasibility study of tVNS device use in individual with MCI. Exploratory secondary analysis will also assess the potential benefits of acute tVNS on cognitive performance. Cognitive function will be assessed under identical conditions in all participants at all three visits and will encompass assessment of the following:

- Face Name Association Task (FNAT): A cross-modal associative memory test [45, 46] hosted on E-PRIME (Psychology Software Tools, Sharpsburg, USA). The participant is shown 30 face-name pairs. After a brief interlude (less than one minute) participants are then shown a combination of faces, some of which have already been displayed. The assessment involves both accurate recognition of recently displayed faces and correct recall of associated names. tVNS has been shown to boost associative memory as assessed by this task in CU older adults [47] but has not been assessed in those with MCI.
- Sustained Attention to Response Task (SART): A computer based response inhibition task [48] hosted on E-PRIME (Psychology Software Tools, Sharpsburg, USA) This is similar to Go/No-Go or Stop/Signal tasks whereby participants are required to withhold response to a single, infrequent target (the digit 3) presented amongst a background of frequent non-targets (0–2, 4–9) to which the participant does respond by pressing the space key on the computer keyboard.
- SeaHero Quest ™ the electronic tablet-based app Sea Hero Quest (GLITCHERS © London, UK; University College London, London, UK) will be used whereby participants will be asked to complete 5 levels of increasing complexity of a nautical-themed navigation task designed to assess speed and accuracy of navigating a boat to a map target [49, 50].
- Language assessments will include list learning, semantic fluency, list recall and list recognition assessments adapted from RBANS battery as tVNS has demonstrated benefits in these domains in both healthy [26, 51] and clinical populations [11, 12].
- Working memory will be assessed via digit span forward and backward which will be undertaken as part of the adapted RBANS battery.

Participants will wear the portable NIRS analysis device during all cognitive tasks so that the TSI may be measured continuously during cognitive tasks at baseline, during active and during sham stimulation.

#### Potential effects of acute tVNS on heart rate variability

HRV assessment will be undertaken using a 3 – lead continuous ECG measure via the Finapres© device. HRV analysis will be undertaken during five minutes of rest pre-active stand or any cognitive assessments. Various HRV measures will be taken, however specifically the root mean square of successive R-R interval differences (RMSSD) is of interest as it has been shown to be most highly correlated with cognition and executive function on metanalysis [52].

#### Potential effects of acute tVNS on inflammatory markers

**Fig. 2:**
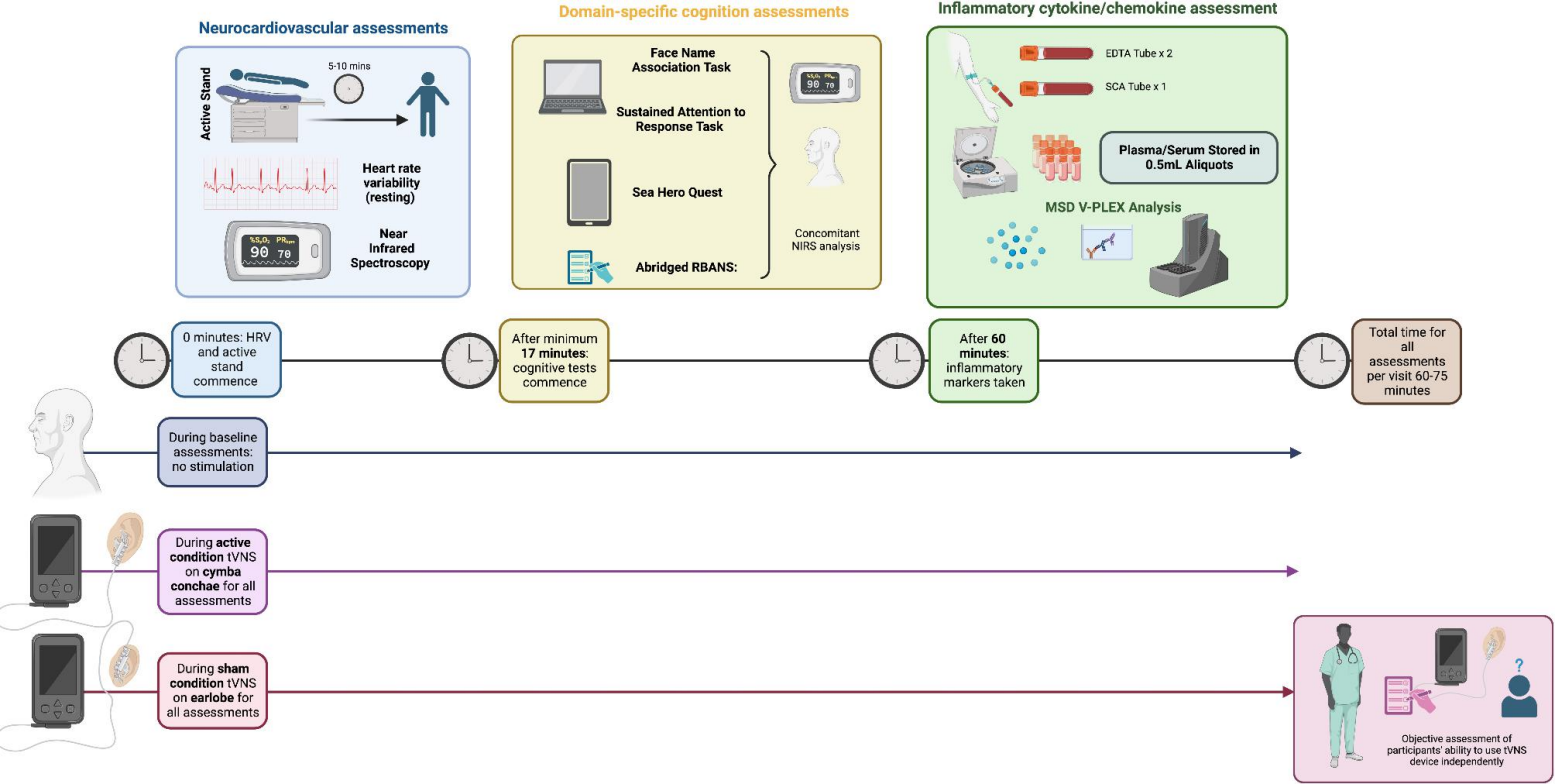
Study visit schedule and design

Aseptic peripheral venepuncture will be performed 50–60 min following neurocardiovascular and cognitive assessments have been undertaken at all three study visits. 7-10ml of blood will be taken in EDTA Vaccutainers, centrifuged at 3,400 rpm for 10 min at 18 °C and supernatant stored in 1ml aliquots at -80 °C for later analysis. A custom-battery of 12 cytokines and chemokines will be analysed using the MesoScale Discovery (MSD) V-PLEX Electrochemoluminescence assay according to standard protocols and includes: IL-1RA, IL-6, IL-8, IL-10, IL-17 A, MIP-1 β, IL12p70, IP-10, TNF- α, MCP-1, Eotaxin, IFN- γ based on known associations with MCI in international literature [53, 54]. Please see Fig. 2 for study visit schedules including minutes of stimulation, methods of assessment and timelines.

### Randomisation

Patients who provide informed consent to participate and who fulfil eligibility criteria will be enrolled into the study. Initial visit (visit 1) involves baseline assessments of neurocardiovascular, cognitive, and inflammatory status. Participants are then randomised to either active or sham stimulation condition at the following visit and then to the alternate condition at the final visit. Participants are blinded to the active or sham stimulation condition but study researchers are not (i.e. single-blinded). Please see Fig. 1 for randomisation strategy.

### Sample size

To establish the safety and feasibility of tVNS in a population of older adults with MCI, VINCI-AD aims to recruit 40 participants for inclusion. This sample size is based on previous literature which investigated the effects of tVNS in healthy young adults [19–21, 23, 55] and also CU older adults [47]. Potential effect sizes from the results of this feasibility study will inform an accurate power calculation for future definitive trials.

### Statistical analysis

VINCI-AD will assess the safety and feasibility of tVNS in older adults with MCI. All participants will be included in final analysis in an “intention-to-treat” fashion. Statistical analysis will be conducted using MATLAB (The MathWorks, Inc., Natick, MA, USA), SPSS (IBM SPSS Statistics for Windows, Armonk, NY: IBM Corp) and GraphPad Prism (GraphPad Software, Inc., San Diego, CA, USA). We anticipate that TSI analysis will require cleaning to remove “noisy” channels i.e. removing large spikes (changes in oxy-Hb larger than 0.5 mM/mm in amplitude); noisy channels will be excluded from analyses and data analyses will utilise software Platform for Optical Topography Analysis Tools [56] with a bandpass filter with cut-off frequencies of 0.01–0.8 Hz to remove instrumental or physiological noise [57].

In the first instance, descriptive statistics will be described for the cohort, reported as means (with standard deviations) or medians (with inter-quartile ranges) and proportions (percentages) as appropriate.

The primary aim of the current study is to assess the safety and feasibility of tVNS in MCI. To assess safety, a chi-square test will compare the number of adverse events reported during the active vs. sham condition. Further safety assessment will elicit symptoms experienced during active stand (using a chi-square statistic), peripheral (beat-to-beat blood pressure response to orthostasis – using mixed-effects linear regression with study condition as the independent variable, with random intercepts for study participant) and central (TSI – also assessed using a linear mixed-effects model). Feasibility will be reported via recruitment rates, successful enrolment, and study completion in proportions and percentages. Tolerability will be assessed using Likert scales, which will be compared between active and sham conditions using paired between-condition statistics (t-test if normally distributed or non-parametric equivalent). Descriptive statistics will be used to report acceptability using the modified Kettle test.

Exploratory secondary aims of this study (cognitive testing, HRV analysis, inflammatory responses) will be analysed using a repeated three – way analysis of variance (ANOVA) if parametric or Kruskal-Wallis test if non-parametric analysis required, with Bonferroni corrections for multiple comparisons. We will explore the effects of concentrations of pro-inflammatory plasma cytokines on cognitive and neurocardiovascular characteristics. Missing data will be handled by applicable statistical methods including multiple imputation.

### Data collection and data management

Data will be collected by research personnel, all of which have been trained in the principles of good clinical practice. Experimental data is pseudo-anonymised then recorded and managed on a secure encrypted local database. Filled-out data collection files will be transferred to the secure database by one researcher. Digitalised data are stored for 5 years after study end. For each participant, a physical Case Report Form, containing all physical source material, will be kept in a secure and locked location for 5 years. Serum samples will be stored at the research laboratory for a maximum of 5 years. Samples will be anonymised and labelled with subject randomisation ID and date of sample collection.

## Discussion

There are limited published studies investigating the efficacy of tVNS in cognition [58] and notably assessments of neurocardiovascular safety of device treatment in this population are lacking. This study investigates the safety and feasibility of a novel handheld neural stimulation device in an older population with memory impairment. Older adults with mild memory impairment are often not included in clinical or device trials [59] despite evidence that older adults with chronic conditions are willing and capable of engaging in digital self-management, and that this cohort can develop and master the technical skills necessary to do this [60]. To our knowledge no formal studies into the safety and tolerability of these devices in a population with cognitive diagnoses has been completed, and most trial designs have not included both baseline and active/sham stimulation arms to control for unblinding bias for the condition. According to findings from a meta-analysis that synthesised all reported adverse events, the safety profile of non-invasive vagal stimulation is excellent [42].

This study has the following strengths. Participants undergo repeated assessments at three time points which will support reliable inference of any effects at statistical analysis. There is also no age limit on this study’s eligibility criteria which is important, as upper age limits in particular have historically excluded older adults from many interventional trials thus limiting generalisability of results. Limitations of this study include planned recruitment of participants from a clinical population to repeated assessments in a clinical research setting which may be challenging in an older cohort with other medical or lifestyle commitments.

In this pilot intervention study we aim to generate clinical, digital and laboratory data to assess to safety, tolerability and potential efficacy for tVNS in a population with amnestic MCI. Further outcomes will include metrics to design future definitive intervention studies, and explore the associations between vagally-mediated neurocardiovascular, inflammatory and cognitive function in this population.

## Electronic supplementary material

Below is the link to the electronic supplementary material.

Supplementary Material 1

Supplementary Material 2

Supplementary Material 3

Supplementary Material 4

Supplementary Material 5

## Data Availability

Not applicable. Data will be pseudo-anonymised using participant unique identification codes assigned at study enrolment and entered to a secure data system on locally-hosted servers for analysis. HD and SK will have access to the secured trial dataset. Data will be published in relevant peer-reviewed journals.
