# Supplementary material for: Safety and feasibility of transcutaneous vagus nerve stimulation in mild cognitive impairment: VINCI-AD study protocol
Source: BMC Neurol. 2023 Aug 2;23:289. doi: 10.1186/s12883-023-03320-5 (PMC10394887; doi:10.1186/s12883-023-03320-5)
Supplement: Supplementary file 1 — Supplementary Material 1 [file 12883_2023_3320_MOESM1_ESM.pdf]

Age Related Memory Service

PTID:__________

Date:__________

Time:__________

Baseline assessment

Sex:

Age:

Height:
Weight:
BMI:

MNA:
Gait speed (TUG or m/s):
Medications:

- __________________
- __________________
- __________________
- __________________
- __________________
- __________________
- __________________

Cognitive history:
Months since testing (___/12)

- RBANS scores
  - Immediate memory:
  - Visuospatial/ constructional:
  - Attention
  - Language
  - Delayed memory:
- EXIT
  - Score
- FAB
  - Score
- CDR
  - Global
  - Sum of boxes
- MMSE:

Clinical background:

- __________________
- __________________
- __________________
- __________________

Charleston Comorbidity index :

- MOCA:
- CBIR
  - Memory orientation:
  - Everyday skills:
  - Self care:
  - Abnormal behaviours:
  - Mood:
  - Beliefs:
  - Eating habits:
  - Sleep:
  - Stereotypic and motor behaviours:
  - Motivation:

Neuroimaging
MTA score:
Fazekas score:
Koedanz score:

FDG PET result:

CSF biomarker:

Total tau _____pg/ml
AB-42 ________pg/ml
p-tau ________pg/ml
