# Supplementary material for: Safety and feasibility of transcutaneous vagus nerve stimulation in mild cognitive impairment: VINCI-AD study protocol
Source: BMC Neurol. 2023 Aug 2;23:289. doi: 10.1186/s12883-023-03320-5 (PMC10394887; doi:10.1186/s12883-023-03320-5)
Supplement: Supplementary file 2 — Supplementary Material 2 [file 12883_2023_3320_MOESM2_ESM.pdf]

Age Related Memory Service

PTID:__________

Date:______________

Time:__________

Assessment of Tolerability of tVNS (active or sham)

Please rate the following on a scale of 1 to 5, 1=no pain, no discomfort 5 = very uncomfortable, intolerable

1. **Are you experiencing any pain?**

   1= no pain 2 = slight pain 3= moderate pain 4= heavy pain 5= severe intolerable pain

2. **Are you experiencing any burning?**

   1= no burning 2 = slight burning 3= moderate burning 4= heavy burning 5= severe intolerable burning sensation

3. **Are you experiencing any tingling sensation?**

   1= no tingling sensation 2 = slight tingling sensation 3= moderate tingling sensation 4= heavy tingling sensation 5= severe intolerable tingling sensation

4. **Are you experiencing any headache?**

   1= no headache 2 = slight headache 3= moderate headache 4= heavy headache 5= severe headache

5. **Are you experiencing any tinnitus or ringing in the ear?**

   1= no tinnitus 2 = slight tinnitus 3= moderate tinnitus 4= heavy tinnitus 5= severe tinnitus

Any other symptoms:

____

____
