# Supplementary material for: Safety and feasibility of transcutaneous vagus nerve stimulation in mild cognitive impairment: VINCI-AD study protocol
Source: BMC Neurol. 2023 Aug 2;23:289. doi: 10.1186/s12883-023-03320-5 (PMC10394887; doi:10.1186/s12883-023-03320-5)
Supplement: Supplementary file 3 — Supplementary Material 3 [file 12883_2023_3320_MOESM3_ESM.pdf]

Age Related Memory Service

PTID:__________

Date:______________

Time:__________

Assessment of Usability of tVNS NEMOS device (active or sham)

Please rate the following on a scale of 1 to 5

1. **Was the device comfortable?**

   1= very comfortable 2 = comfortable 3= neutral 4= moderately uncomfortable 5= severe uncomfortable

2. **Did you feel confident using the device?**

   1= very confident 2 = mostly confident 3= neutral 4= not confident 5= lost, would not know what to do with it

3. **Would you use it again?**

   1= yes without hesitation 2 = probably 3= maybe 4= probably not 5= definitely not

Any other symptoms:
____

____
