# Supplementary material for: Safety and feasibility of transcutaneous vagus nerve stimulation in mild cognitive impairment: VINCI-AD study protocol
Source: BMC Neurol. 2023 Aug 2;23:289. doi: 10.1186/s12883-023-03320-5 (PMC10394887; doi:10.1186/s12883-023-03320-5)
Supplement: Supplementary file 4 — Supplementary Material 4 [file 12883_2023_3320_MOESM4_ESM.pdf]

Age Related Memory Service

PTID:__________ Date:______________ Time:__________

tVNS Functional Assessment

Scoring & Comments on task performance

Score > Step	0 Intact	1 trial & error	2 general cue	3 a. specific cue	4 assisted
1. Taking tVNS device out of zip bag					
2. Identifying ear piece, cord, liquid, plastic covers and stimulator					
3. Identifying input area for cord into tVNS stimulator					
4. Manually inserting cord into tVNS stimulator the right way up					
5. Turning on tVNS device using correct button					
6. Choosing ear plug that will fit their ear					
7. Placing small plastic covers on electrodes					
8. Rub earpiece in tVNS stimulator liquid					
9. Placing ear piece into correct ear and correct part of ear					
10. Appropriately pressing correct button to increase tVNS stimulation parameters					
11. Appropriately troubleshooting common problems (device loses contact with skin or falls out of ear)					
12. Choosing correct tVNS stimulation parameters (below pain threshold)					
13. Disassembling tVNS device appropriately and safely					
**Total score = /52**	(	+	+	+	+)

Note: If a cue was given, indicate why and what cue was given.

Scoring: Total score 0-52, higher scores reflect more severe problems in performance.

0 **Intact performance**

1 **Slow and/or trial & error**, and/or questionable performance, but completes independently

2 Received **general cues**
(that does not give specific information about the performance or details of the task. Usually, in the format of a general question, such as:
"what do you have to do now?"; "what is the next step?"; "what else do you need?" etc.)

3 Received **specific cueing**
(that guides the client to take specific steps or delineates details of the task:
"now you have to turn on the device", "is the stimulation on", "its not currently stimulating")
OR **incomplete performance ie** turns on device but forgets how to increase parameters, puts in ear before turning it on

4 **Received physical demonstration or assistance**, such as demonstrating how to connect the electric cord to the stimulator, or assisting the client in turning on the device.

tVNS Test Protocol

Materials:

- tVNS device –disassembled parts including device, connector cord, stimulation liquid, electrode covers, charger

Before starting the test:

Ask the client to repeat the instructions until you verify that he/she knows the details of the task and understands them. ☐
If he/she gets it wrong, repeat the instructions. Don't start until the client can repeat the instructions correctly (unless the client has a documented language disorder).

During the task you (the examiner) stand by within reaching distance from the client for safety precautions (close enough for intervention if necessary).

Observe the client's performance without any cueing or intervention (verbal or physical) unless one of the following situations occur:

- **Performance is unsafe for the client or the environment** (for example, mishandling electricity, unsafe usage of device) ☐
- **Task progression is stopped**: client does not give any indication of action for more than a minute ☐
- **Client demonstrates repeated failure** or requests assistance and is unable to progress independently with task steps ☐

Cueing Procedure - if required (for situations listed above)

2. General cue
3. Specific cue
4. Physical demonstration or assistance

Reference: Hartman-Maeir A, Armon N & Katz N. (2005) The Kettle Test: A Cognitive Functional Screening Test protocol.
